# Need for NAMs: A systematic evidence synthesis revealing over half a century of drug development failure

**DOI:** 10.1016/j.namjnl.2026.100119

**Published:** 2026-07-21

**Authors:** Dilyana Filipova, Wolfgang Boomgaarden, Leyla Fox, Andrew Knight, Bettina Lickiss, Christian Maass, Gaby Neumann, Justine Watkins, Kathrin Herrmann, Lorna Ewart, Matthias Gossmann, Merel Ritskes-Hoitinga, Tamara Zietek

**Affiliations:** a Doctors Against Animal Experiments (DAAE), Lustheide 85, 51427, Bergisch Gladbach, Germany; b European Coalition to End Animal Experiments (ECEAE), Lustheide 85, 51427, Bergisch Gladbach, Germany; c PharmaInformatic, Nesserlander Str. 92, 26723, Emden, Germany; d School of Veterinary Medicine, College of Environmental and Life Sciences, Murdoch University, Perth, 90 South St, Murdoch WA 6150, Australia; e School of Environment and Science, Griffith University, Brisbane, 170 Kessels Road, Nathan QLD 4111, Australia; f Faculty of Health and Wellbeing, University of Winchester, Sparkford Road, Winchester, Hampshire, SO22 4NR, UK; g innoVitro GmbH, Artilleriestr. 2, 52428, Jülich Jülich, Germany; h ESQlabs GmbH, MPSlabs, 26683, Saterland, Germany; i Faculty of Veterinary Medicine, Utrecht University, Heidelberglaan 8, 3584 CS, Utrecht, Netherlands; j Center for Alternatives to Animal Testing (CAAT), Johns Hopkins Bloomberg School of Public Health, 615 N. Wolfe Street, Baltimore, MD, 21205, USA; k Emulate, Inc., 27 Drydock Avenue, Boston, MA, 02210, USA; l Aarhus University, Nordre Ringgade 1, 8000, Aarhus C, Denmark

**Keywords:** Drug attrition, Success rate, Likelihood of approval, Drug development, Reasons for drug failure

## Abstract

**Background:**

Preclinical assessment of drug safety, efficacy and pharmacokinetics still relies largely on animal‑derived data, despite their limited ability to predict human outcomes. New Approach Methodologies (NAMs) are emerging as more human‑relevant preclinical tools. This systematic evidence synthesis examines (1) current levels and temporal trends in clinical drug attrition and (2) the proportion of failures attributed to biological factors such as safety, efficacy and pharmacokinetics that are routinely evaluated during preclinical testing, to identify priority areas for NAM implementation.

**Methods and findings:**

We systematically searched PubMed on March 5, 2024, for peer-reviewed, English-language studies since 1963 reporting numerical primary data on clinical drug attrition rates and/or reasons for failure. The protocol was prospectively registered (osf.io/46mw5). We excluded non-original research and studies on specific cases, diseases, or single drug classes. Thirty-two publications met the criteria, yielding 60 attrition and 36 failure-reason datasets (1963–2017). Across six decades, mean clinical attrition ranged from ∼80 to ∼91 % with the highest rates within the past two decades. Overall, biological factors (safety, efficacy, pharmacokinetics) accounted for ∼77% of reported causes. Limitations include heterogeneous reporting, restriction to English, and exclusion of grey literature.

**Conclusions:**

Clinical drug attrition has not improved in the last six decades, peaking at ∼91% in the 2000s–2010s, with most failures linked to biological factors. These findings suggest that prevailing preclinical approaches – many of which heavily rely on animal models – often have limited ability to predict clinical outcomes and highlight the urgent need for more predictive, human-relevant NAMs.

## Highlights

• First synthesis of six decades of clinical drug attrition and reasons for failure.
• Clinical attrition at ∼80–91% since the 1960s, peaking at ∼91% in the 2000s–2010s.
• 77% of failures due to biological factors, highlighting limits of animal testing.
• Findings support the need for human-relevant NAMs in preclinical drug assessment.

## 1 Introduction

Success rates in drug development are notoriously low, with fewer than 1 out of 10 drug candidates that enter clinical trials gaining marketing approval (Dowden and Munro, 2019; Thomas et al., 2021). To understand these low success rates, it is crucial to consider the complex nature of drug development. The process is multi-staged, progressing from discovery to clinical application. Following initial identification, drug candidates undergo rigorous preclinical evaluation, culminating in toxicity and efficacy tests across multiple animal species (Deore et al., 2019). Successful candidates then advance to human clinical trials (phases I-III) involving both healthy volunteers and patients (Deore et al., 2019). This lengthy and intricate process contributes to high attrition rates, which in turn push up research and development (R&D) times and costs. On average, pharmaceutical companies need more than 13 years and up to 6 billion US dollars to successfully develop one drug, with roughly 75% of R&D cost resulting from drug attrition (Paul et al., 2010; DiMasi et al., 2016; Rennane et al., 2021; Lo and Field, 2009). In addition, a growing number of animal trials are conducted in basic and preclinical research, with one study estimating a 37% increase in animal use for research purposes worldwide from 2005 to 2015 (Taylor and Alvarez, 2019). Interestingly, a 2024 analysis of European statistics estimated that animals used by the pharmaceutical industry for drug development account for about 20% of all animals used for scientific procedures in Europe, down from roughly 30% in 2005, despite increasing research spending (Hartung, 2024). This reduction likely reflects a combination of factors, including increased use of new approach methodologies (NAMs), more refined and ethically oriented animal study designs, and broader adoption of weight-of-evidence approaches in regulatory frameworks (e.g. ICH S1B for carcinogenicity and ICH S5 for reproductive toxicity), which allow more tailored, data-driven decisions about when long-term animal studies are needed (FDA, 2022, 2021).

We recognize that the term “NAMs” is used variably in the literature and acknowledge the need for a unified term (LaFollette et al., 2026). Here, in the context of drug development for human use, we use it as an umbrella term for non-animal, human-relevant methods and strategies, including *in vitro, in silico* and other emerging approaches, that aim to improve the prediction of human biological responses in preclinical assessment and related biomedical research stages. NAMs such as human organoids, organs‑on‑chips, and advanced computational models are being developed to generate more human‑relevant data and are attracting growing interest as more predictive preclinical tools in drug development (Ewart et al., 2022; Mirlohi et al., 2025). This momentum is reflected in regulatory initiatives across jurisdictions that explicitly encourage or permit the use of NAMs in place of animal tests, including the U.S. FDA Modernization Act 2.0 and the European Commission’s recently published Roadmap towards phasing out animal testing for chemical safety assessments (Zushin et al., 2023; European Commission, 2026). Together, these trends underscore the need to understand where traditional animal-based approaches fall short and where NAMs could improve clinical translation.

Proof of drug safety and efficacy in animals has traditionally been required by regulatory agencies prior to clinical trials (EMA, 2009; FDA, 2010). Animal-derived data are intended to play a decisive role in determining which drug candidates will proceed to first-in-human studies, yet several reports have criticized the utility and quality of these preclinical data (Sievers et al., 2021; Wieschowski et al., 2019). To define how well NAMs must perform to be suitable for drug testing, it is first essential to understand how effective classical, animal-based approaches have been to date by examining long-term trends in clinical drug attrition and the extent to which safety, efficacy, and other biological parameters typically assessed in animals contribute to these failures.

While similar disease mechanisms exist in humans and animals, and some drugs are used in both human and veterinary medicine, this does not by itself demonstrate that animal models are reliable predictors in human drug development (Scott et al., 2020). Biological and pharmacological interspecies differences are often cited as a major reason for poor translatability of animal data to humans, leading to unreliable preclinical drug safety and efficacy evaluation (DeKeyser and Shou, 2012). Significant differences between human and animals have been demonstrated in various aspects, including drug uptake, oral bioavailability, and toxicity responses (Zietek et al., 2020, 2021; Van Norman, 2019). For instance, preclinical animal trials have shown limited ability to predict human toxicities in Phase I oncology clinical trials, and one analysis estimated that only 19% of serious human adverse effects are identified in animals (Atkins et al., 2020; van Meer et al., 2012). A recent review highlights how poor translatability of animal-derived data has hindered drug development in several major disease areas (Marshall et al., 2023). These interspecies differences likely contribute substantially to high drug failure rates and highlight the need for more human-relevant NAMs. Systematically analyzing trends in drug attrition and their underlying causes could help identify areas where available NAMs could already deliver better predictive performance.

Over the past 60 years, numerous regulatory, scientific and industry developments have influenced clinical drug attrition. A key milestone was the 1962 Kefauver-Harris Amendments to the Federal Food, Drug, and Cosmetic Act in the United States, which required proof of both safety and efficacy and explicit FDA approval before marketing, thereby raising standards for pharmaceutical R&D and manufacturing (Greene and Podolsky, 2012). Subsequent legislation and guidance further tightened production quality and approval criteria, potentially making market authorization more difficult (Meadows, 2019). On the other hand, some argue that more demanding regulatory requirements force pharmaceutical companies to be more selective with their drug candidates, which in turn raises their likelihood of approval (Munos, 2009). Further changes that have affected drug development and attrition during the last six decades include diverse measures introduced for the protection of human subjects in clinical trials, changes in clinical trials’ design, improvements in standard-of-care treatments, and involvement of new technologies like big data-based approaches and precision medicine, among others (FDA, 2018; Katz, 2004; Li and Bergan, 2020; Bhutta, 2004). Fluctuations in failure rates in preclinical trials and the expansion of the scope of preclinical evaluation may also affect drug candidates’ probability of success (Hop, 2015). These and other drivers of attrition have been the subject of multiple investigations and are thus not the focus of the present study (Munos, 2009; Takebe et al., 2018; Lawton, 2015; Clay, 2015; Van Norman, 2019). Instead, our aim is to systematically map and summarize published evidence on clinical drug failure rates and their main reported causes from 1963 onward, to provide an overview of trends and knowledge gaps and to inform future, more targeted analyses of specific contextual factors and the potential role of NAMs in reducing attrition. Nevertheless, one should keep in mind that all of the above-mentioned factors may have impacted the past and current state of drug attrition.

Multiple scientific publications and industry reports have examined drug approval and attrition, but most cover only limited time windows (typically 3–10 years) and focus on specific clinical phases, disease areas, or drug modalities, often embedded within broader analyses of R&D strategy and performance. As a result, extracting comparable data and reconstructing long-term trends in clinical failure is labor-intensive, and a single, synthesized estimate of overall attrition is rarely available. To address this gap, our systematic evidence synthesis collates and summarizes published evidence on clinical drug failure rates and their main reported causes from 1963 onward, providing an integrated view of trends over six decades and a foundation for future work on how evolving approaches – including NAMs – might reduce attrition.

In contrast to prior reviews on narrow time frames, individual clinical phases, or specific drug classes, this systematic analysis offers the first comprehensive synthesis of clinical drug attrition rates and reported reasons for failure across nearly six decades of pharmaceutical development. We aggregate failure rates and systematically categorize the underlying causes, providing a longitudinal perspective that has been missing in the literature. Although we do not systematically analyze regulatory or industry changes, we discuss our findings in the context of major developments – including the rapid rise of NAMs – to underscore the contemporary relevance of observed attrition patterns.

In this systematic evidence synthesis, we seek to answer the following two research questions:

**RQ1: Clinical drug failure:** What percentage of drug candidates that enter clinical Phase I fail to obtain a marketing authorization, and what trends can be detected in these numbers within the last six decades, i.e. from 1963 to date?
**RQ2: Reasons for failure:** What percentage of drug candidates fail between Phase I clinical trials and drug authorization for reasons related to unsatisfactory drug safety, efficacy, bioavailability and pharmacokinetics (PK), or other factors classically evaluated in animals during preclinical drug assessment (henceforth described as “biological reasons”) compared to economic, strategic, and commercial reasons (henceforth described as “non-biological reasons”)?

Both research questions focus exclusively on reported values from clinical stages of drug development – encompassing clinical trial phases I-III, new drug application (NDA) submission, and approval – while excluding data related to preclinical development.

We address these questions by performing a systematic search of the peer-reviewed literature on PubMed. Our analysis spans from 1963, the year following the implementation of the Kefauver-Harris Amendments, to the present day, mapping relevant data for both research questions across this pivotal period in pharmaceutical regulation and development.

## 2 Methods

**Fig. 1:**
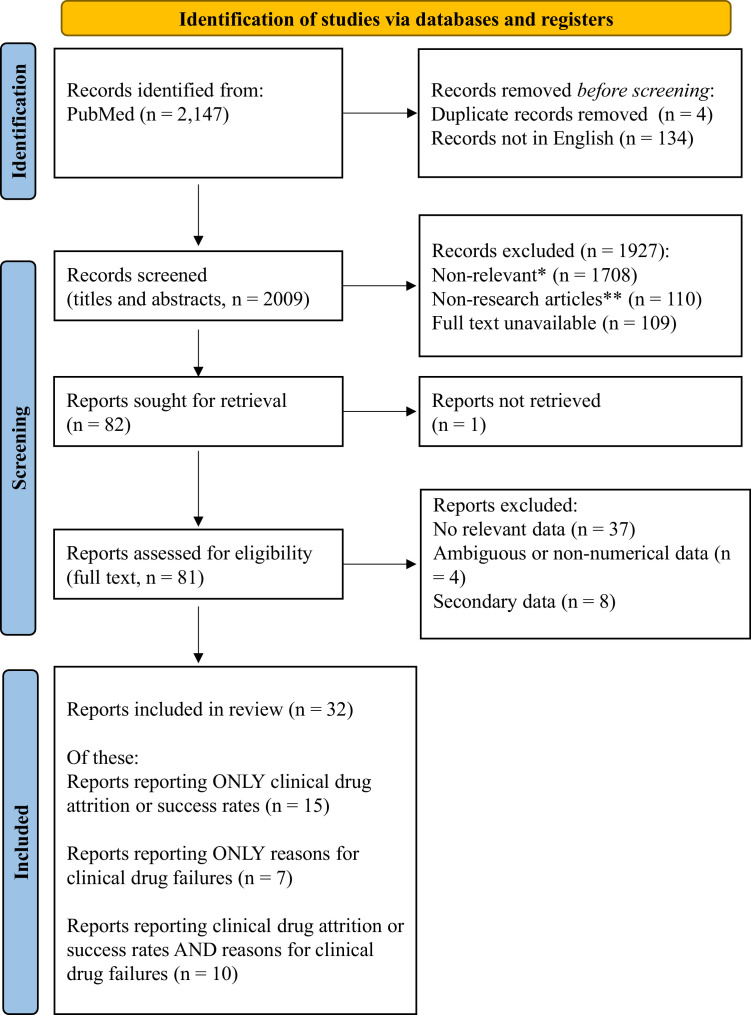
Adapted preferred reporting items for systematic reviews (PRISMA) flow diagram. The diagram indicates the search and screening process for the present systematic evidence synthesis. *Non-relevant publications include those outside of the field of drug development, case studies, preclinical studies, publications focused on ethical, legal and societal drug development topics, publications analysing only approved drugs, as well as studies focused on specific diseases, specific molecular drug classes, examining the properties of a single or a few compounds, etc. **Non-research articles include corrigenda, commentaries to publications, letters, editorials, news articles, regulatory articles.

This systematic evidence synthesis was conducted in accordance with the Preferred Reporting Items for Systematic Reviews and Meta-Analyses guidelines for scoping reviews (PRISMA-ScR, Fig. 1, Supplementary Tables S1 and S2) (Tricco et al., 2018). The protocol was prospectively registered on the Open Science Framework (OSF) platform (project: osf.io/46mw5; protocol workflow: osf.io/m6xn7).

### 2.1 Search strategy and eligibility criteria

To obtain an overview of the scientific literature, we searched PubMed for terms related to drug attrition, drug success rates, and reasons for failure. We restricted our search to PubMed for two reasons. First, PubMed offers extensive coverage of biomedical literature, providing access to more than 37 million citations from MEDLINE, life science journals and online books, and is widely used for systematic searches. Second, due to resource constraints and lack of institutional access to subscription based databases such as Embase and Web of Science, we were unable to include additional databases in this analysis. As data on drug attrition is often provided within the context of evaluating the drug development cost and economic parameters, especially when analyzing the pharmaceutical industry in the United States, we included potentially relevant economic terms in our search strategy. Following an initial scoping trial, we employed the following search strategy, utilizing a combination of keywords within MeSH terms [mh] and title fields [ti] as appropriate: (Drug Evaluation, Preclinical [mh] AND economics [mh]) OR (Drug Industry [mh] AND Product Surveillance, Postmarketing [mh] AND methods [mh]) OR (clinical [ti] AND success [ti] AND rates [ti]) OR (attrition [ti] AND rates [ti]) OR (“Drug Industry” [mh] AND “United States” [mh] AND “Time Factors” [mh]) OR (phase [ti] AND failures [ti]) OR (Drugs, Investigational [mh] AND United States [mh] AND Time Factors [mh]) OR (Drugs, Investigational [mh] AND Drug Industry [mh]) OR (Drugs, Investigational [mh] AND economics [mh]) OR (Drugs, Investigational [mh] AND clinical [ti] AND Failure [ti]) OR (Drugs, Investigational [mh] AND success [ti] AND rates [ti]). The search was performed on March 5, 2024.

To ensure the inclusion of high-quality and relevant data, the following eligibility criteria were established for this systematic evidence synthesis:

1. **Peer-reviewed publications**: Only peer-reviewed publications were considered for inclusion. This criterion was set to maintain a consistent standard of quality and reliability, avoiding the variability often associated with non-peer-reviewed sources.
2. **Exclusion of non-research articles**: We excluded corrigenda, commentaries, letters, editorials, news articles, regulatory articles, and similar non-research articles. These types of publications do not typically contribute primary research data and are therefore not aligned with the objectives of this review.
3. **Primary literature only**: The review focuses exclusively on primary literature. As such, review articles were excluded to ensure that the analysis is based on original research findings.
4. **Language**: Only publications written in English were screened and analyzed. This language restriction is applied to ensure the feasibility of comprehensive analysis and interpretation.

By adhering to these criteria, the review aims to provide a robust and focused synthesis of the available primary research literature within the scope of the research questions (RQ1 and RQ2).

After the search was completed, duplicates and publications not written in English were excluded from further analysis. Subsequently, the titles and abstracts of the remaining studies were screened, whereby non-primary research and publications, for which the full text was unavailable, were excluded. Furthermore, studies were classified as non-relevant and excluded from the subsequent analysis if they met one or more of the following criteria:

1. **Scope:** Publications outside the field of drug development were excluded.
2. **Study design**:
  • Case studies were considered non-relevant.
  • Preclinical studies were excluded.
3. **Topic focus**:
  • Publications primarily focused on ethical, legal, or societal aspects of drug development were not included.
  • Studies analyzing only approved drugs were deemed non-relevant.
  • Publications examining specific diseases or specific molecular drug classes were excluded.
  • Studies focused on the properties of a single compound, or a limited number of compounds were not considered relevant.
4. **Specificity**: Any publications that were too narrow in focus and did not contribute to the broader understanding of drug development processes were classified as non-relevant.

The full text of the remaining records was sought for retrieval. The following additional eligibility criteria were applied to the remaining records, specific to research questions 1 (RQ1) and 2 (RQ2):

**Criteria applicable to both RQ1 and RQ2:**

1. **Numerical data**: The text must include numerical values (either absolute numbers or percentages) pertaining to:

• Drug candidate attrition/success rates (RQ1)

and / or

• Reasons for failure (RQ2)

2. **Data originality**: The relevant data must be original to the publication and not cited from previous studies.
3. **Separation of preclinical and clinical data**: If preclinical drug attrition/success rates or reasons for failure are included, these must be clearly distinguishable from the clinical drug attrition/success rates and reasons for failure.

**Criteria specific to RQ1:**

4. **Comprehensive clinical trial coverage**: The reported values must encompass all stages of clinical drug testing, from phase I through the end of phase III, NDA submission, or drug authorization. This ensures a complete representation of the entire clinical trial period.

**Criteria specific to RQ2:**

5. **Flexible phase coverage**: The reported values can either:

• Encompass all stages of clinical drug testing (from phase I through the end of phase III, NDA submission, or drug authorization), OR
• Reflect the reasons for failure for one or several specific clinical phases.

Two reviewers screened the full-text publications, evaluated whether each record met all eligibility criteria and charted the data independently from each other. Disagreements were resolved by internal discussion.

### 2.2 Data charting and analysis

A data-charting form was developed and used by two reviewers to extract the relevant variables. All records were screened and data were extracted manually using standardized spreadsheets; no dedicated review management software was used. The two reviewers independently charted the data, discussed the results and updated the data-charting when necessary. For each eligible publication, the following data were extracted: PubMed identifier (PMID), title, authors, analysis period (in years, e.g. from 1963 to 1966), data source (e.g. database, survey, etc.), clinical phases analyzed, clinical attrition rate (if applicable) and reasons for clinical failure (if applicable).

#### 2.2.1 Clinical drug failure

For the purpose of this work, we define clinical drug attrition as follows: The percentage of drug candidates that enter Phase I clinical trials but fail to obtain marketing authorization, out of the total number of drug candidates entering Phase I clinical trials.

Depending on the type of data provided by each analyzed study, the clinical drug failure was extracted or calculated via one of the following methods:

1. **For publications reporting the percentage of clinical drug attrition:**
  The reported attrition data was taken directly as provided in the publication.
2. **For publications providing concrete numbers of candidates:**
  When publications provided specific numbers of drug candidates entering Phase I and the number of successful candidates, the failure rate was calculated using the following formula:
  Failure rate (%) = [(N _Phase I_ - N _NDA_) / N _Phase I_] × 100

Where:

N _Phase I_ is the number of drug candidates starting Phase I clinical trials
N _NDA_ is the number of candidates successfully obtaining New Drug Application approval
3. **For publications reporting clinical success rates or likelihood of approval:**
  The overall clinical failure rate was calculated using the following formula:
  Failure rate (%) = 100% - Success rate (%)

Where 100% represents all drug candidates entering Phase I clinical trials, and the success rate represents the percentage of authorized drugs.

4. **For publications reporting success rates by clinical trial phase:**
  When publications provided the percentage of drug candidates successfully completing each phase of clinical trials, we calculated the overall clinical success rate by multiplying the likelihood of transition for phases I, II, and III. The overall clinical failure rate was then determined as:
  Failure rate (%) = 100% - (Phase I success % × Phase II success % × Phase III success %)
5. **Special cases:**
  In some instances, the reported clinical failure rate pertains to drug candidates failing between phases I and III, without considering failures at the New Drug Application (NDA) submission/approval stage. We included these data in our analysis, as we anticipated only marginal drug failure at the NDA stage.

#### 2.2.2 Reasons for clinical drug failure

In our study, we analyzed the reasons for clinical drug failure by examining parameters typically evaluated during preclinical trials in animal models. We categorized these reasons into two main types:

1. **Biological Reasons**:
  These reasons pertain to drug candidate properties that are commonly assessed in animals during preclinical trials. They include factors such as:
  • Drug safety / toxicity.
  • Drug efficacy.
  • Bioavailability and pharmacokinetic/pharmacodynamic (PK/PD) properties.
  • Other similar biological factors.
2. **Non-Biological Reasons**:
  These reasons are related to factors beyond the biological properties of the drug candidates. They include:
  • Strategic or economic considerations.
  • Portfolio optimization by the company.
  • Reasons that are undisclosed or listed as "others".

By distinguishing between these categories, we aim to provide a comprehensive understanding of the factors contributing to clinical drug failure, encompassing both biological assessments and broader strategic considerations.

#### 2.2.3 Publications with multiple data points

Some of the identified studies reported multiple relevant data points, which we handled as follows:

1. **Multiple time periods**:
  When a study reported data on clinical drug attrition or reasons for clinical drug failure across multiple time periods, we analyzed the values for each presented period separately.
2. **Multiple sets of drug candidates**:
  Some publications presented relevant data for several distinct sets of drug candidates (e.g., clinical drug attrition in US-owned firms versus non-US-owned firms). In these cases, we treated each set as a separate data point.

For both scenarios described above, each relevant data point was included as a separate value in our analyses. This approach allowed us to capture the full range of reported data while maintaining the integrity of the original studies' findings. It also enabled a more nuanced analysis of trends across different time periods and subgroups within the pharmaceutical industry.

### 2.3 Critical appraisal of individual data sources

To better understand the variability among included studies, such as differences in primary focus, data sources, and dataset descriptions, we conducted a critical appraisal to explore relevant study characteristics and assess the scope of information provided. We developed a quality assessment checklist comprising eight key data items: 1. Attrition rates, 2. Reasons for failure, 3. Originality of data, 4. Defined analysis period, 5. Defined analysis sample, 6. Number of drug candidates, 7. Number of pharmaceutical companies, 8. Type of drug candidates. These items were selected based on their ability to contextualize the reported data values and aid in their interpretation. One point was awarded for the presence of each data item. A quality score was calculated for each study, with a maximum possible score of eight. Lower scores do not necessarily indicate low overall methodological quality but suggest that some information relevant to our research questions was not provided. The quality assessment checklist was incorporated into the data extraction form. Both reviewers independently completed the checklist during the data charting process. This critical appraisal approach allowed us to systematically evaluate the completeness and relevance of the information provided in each study, enhancing our ability to interpret and synthesize the findings in the context of our research questions.

### 2.4 Assessment and management of heterogeneity

Given the anticipated diversity in study periods and reporting, we planned a priori to assess heterogeneity by grouping extracted data into decades (1960s, 1970s, 1980s, 1990s, 2000s, and 2010s) and comparing drug attrition rates and reasons for failure over time. The primary approach to assess heterogeneity by decade was specified a priori in our protocol (see OSF registration). However, some operational decisions, such as assigning studies spanning multiple decades to the decade most represented by the data, were made during the review process to best accommodate the data structure. We used descriptive statistics and visualizations (e.g., box plots) to illustrate temporal trends and variability, and all analyses were conducted using the original, unadjusted data to preserve the integrity of reported findings.

We also considered other sources of heterogeneity, such as differences in study design, sample size, and, importantly, data sources. Over the six decades analyzed, there was a clear evolution from author-conducted surveys to the use of large, proprietary databases. This shift, along with the lack of disclosure of individual drug candidate identities in most studies, limited our ability to precisely assess data overlap between datasets. As a result, some degree of redundancy or variation due to overlapping or non-overlapping data sources is possible, which may have contributed to the observed variability in attrition rates and reasons for failure. To further contextualize observed heterogeneity, we applied a quality assessment checklist to each included study, evaluating key characteristics relevant to data interpretation. As this was an evidence synthesis, our approach was primarily descriptive and did not include formal meta-analytic techniques. Therefore, residual heterogeneity related to unmeasured or unreported study characteristics may persist.

### 2.5 Potential bias and mitigation strategies

We acknowledge that several authors are affiliated with organizations advocating for or developing non-animal research methods, while others are based in academic institutions internationally. To minimize potential bias and ensure objectivity:

• The review protocol, research questions, and eligibility criteria were prospectively registered on the Open Science Framework prior to data collection.
• Study selection and data extraction were performed independently and in a blinded manner by two reviewers, using predefined inclusion and exclusion criteria.
• A custom quality assessment checklist was developed and applied systematically to all included studies, as described in the *Critical appraisal of individual data sources* section.
• All data, extraction forms, and analysis steps are transparently reported and available in the supplementary materials.
• Key findings and interpretations were discussed among all co-authors, representing a range of institutional backgrounds, to ensure balanced reporting.

We believe these measures have minimized the risk of bias and ensured a comprehensive and objective synthesis of the available literature.

### 2.6 Statistics and synthesis of results

**Fig. 2:**
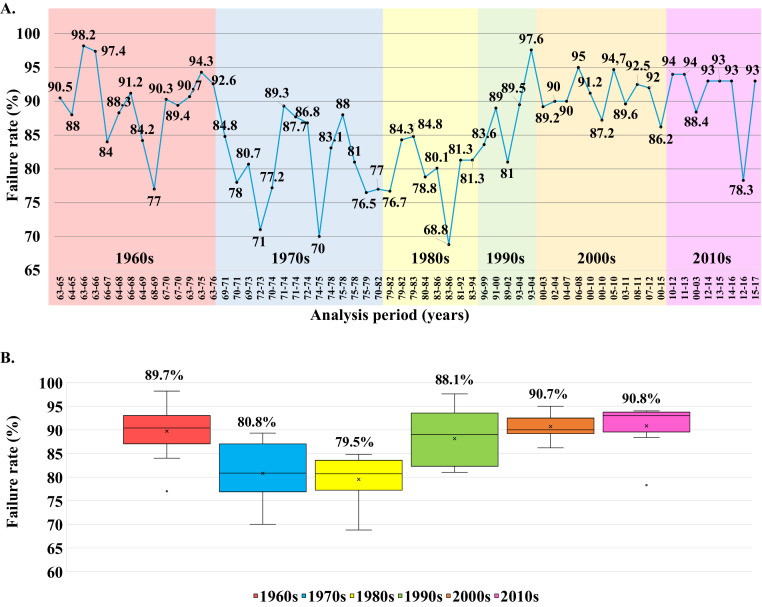
Clinical failure rates from the 1960s to 2010s. (A) Drug candidate attrition trends from 1963 to 2017. Data points represent failure rates for periods indicated on the x-axis (see Supplementary Table S3). Color shadings indicate data points included in each decade's analysis. (**B**) Mean clinical failure rates across six decades. Boxes show exclusive interquartile range (IQR) with median (line), mean (x), and min/max values (whiskers). Numbers above boxes indicate mean failure rates.

Our statistical analysis was performed using Microsoft Excel (version 2501) and focused on two main aspects:

For RQ1, we calculated the mean clinical drug failure rate using the individual values provided in Supplementary Table S3. We extracted drug attrition values from 1963 to 2017 and grouped the appropriate data points into decades (1960s, 1970s, 1980s, 1990s, 2000s, and 2010s) to clearly illustrate the trends of clinical drug failure over time. The box plot in Fig. 2B was generated using these grouped values to visually represent the distribution and trends of failure rates across the decades.

For RQ2, the box plots in Fig. 4 (A and B) were created using the values given in Supplementary Table S4, illustrating the distribution of different reasons for clinical failure.

For Figs. 2B, 4A and B, the box plots display the following:

• Boxes represent the exclusive interquartile ranges (IQRs).
• Median values are indicated by a line within each box.
• Mean values are marked with an 'x'.
• Whiskers extend to the minimum and maximum values.
• Outliers are depicted as individual dots.

It is important to note that the data was not adjusted or normalized in any way. We chose to maintain the original data to avoid potentially skewing the results and to ensure the most accurate representation of the reported findings.

## 3 Results

Our search identified 2,147 citations, of which four duplicates and 134 non-English publications were excluded (Fig. 1). Of the remaining 2,009 titles, a total of 1,927 records were excluded on the basis of being non-relevant (1,708), non-research articles (110) or their full text was unavailable (109). We could not retrieve one of the remaining 82 records. We screened the full text of the 81 remaining records and excluded 37 records that did not contain data relevant to the research questions (RQ1 and/or RQ2), four records containing only ambiguous or non-numerical data, and eight records containing only secondary data. In total, 32 publications met all eligibility criteria. Of these, 15 publications report relevant clinical drug attrition data (RQ1), seven publications indicate reasons for clinical drug failure (RQ2), and 10 publications contain data on both clinical drug attrition and clinical reasons for drug failure (RQ1 and RQ2) (Supplementary Tables S3 and S4).

We critically appraised the robustness of the information relevant to RQ1 and RQ2 in each study included in this analysis by devising a quality assessment checklist comprising eight key data items (Supplementary Table S5). The presence of each data item increased the quality score of the respective study by one point, with a maximum possible score of 8 points. The highest scores were detected in the 10 publications that report data for both clinical drug failure rates and reasons for failure – with seven studies having a score of 8, two studies having a score of 7, and one study with a score of 6 points. Among the 17 publications with data only on the clinical drug failure rates, six had a score of 7, seven had a score of 6, and two had a score of 5 points. From the seven studies that only showed reasons for drug failure, one had a score of 7, three had a score of 6, and another three had a score of 5 points. While this score system does not consider the methodological rigour of the individual publications, the studies with higher scores painted a more detailed picture of the parameters of drug attrition that are the focus of this systematic evidence synthesis.

### 3.1 Clinical failure rate trends from 1963 to 2017

To address research question RQ1, which focuses on the current state and temporal trends of clinical drug failure rates, we analyzed attrition rates of drug candidates entering Phase I clinical trials from 1963 onward. This analysis was based on data reported in 25 publications, as detailed in Supplementary Table S3 and illustrated in Fig. 2A. In total, these studies provided data for 60 partially overlapping periods, each spanning 2 to 16 years. For publications containing drug attrition data across multiple time spans, we listed all failure rates separately, as outlined in the Methods section. The number of investigated drug candidates varied significantly, ranging from 58 to 21,143 compounds. When publications provided the total number of evaluated drugs across several analysis periods, we calculated and presented the mean number of drug candidates per period (Supplementary Table S3). For instance, DiMasi et al. (1994) evaluated success/attrition rates for a total of 1,177 drug candidates across four time periods (1964-1968, 1969-1973, 1974-1978, and 1979-1983), resulting in a mean number of approximately 294 drug candidates per period (DiMasi et al., 1994). This approach allows for a standardized comparison across studies with varying scales and time frames while preserving the integrity of the original data.

Analysis of the 60 drug attrition data points revealed the following distribution: one (1.7%) fell between 60% and 69.9%, 10 (16.7%) between 70% and 79.9%, 27 (45%) between 80% and 89.9%, and 22 (36.7%) between 90% and 100% (Fig. 2A, Supplementary Table S3). The sole value below 70% (68.8%) is derived from a study of only 64 compounds developed by non-US pharmaceutical firms between 1983 and 1986. Notably, the same study reported a failure rate of 80.1% for 201 drug candidates developed by US pharmaceutical companies during the same period, suggesting potential geographical differences in preclinical and/or clinical analysis approaches (DiMasi et al., 1991). Interestingly, both the highest and lowest clinical failure rates in our dataset come from the same publication. The highest rate (98.2%) pertains to 333 New Chemical Entities (NCEs) from US-owned firms between 1963 and 1966. For the same period, the study reports a 97.4% attrition rate for 78 NCEs from non-US-owned firms. This close alignment in failure rates between US and non-US firms in the 1960s contrasts sharply with the divergence observed in the 1983-1986 period, highlighting potential shifts in drug development practices or success rates across different geographies and time frames.

The earliest failure rate in our dataset describes the attrition of 211 compounds evaluated in clinical trials between 1963 and 1965, with a calculated failure rate of 90.5% (Sheck et al., 1984). At the other end of the timeline, the most recent data examines compounds developed by 30 pharmaceutical firms from early 2015 to the end of 2017, yielding a mean failure rate of 93% (Dowden and Munro, 2019).

To more clearly observe drug attrition trends over time, we calculated mean failure rate values for each decade from the 1960s (starting in 1963) to the 2010s (until 2017) (Table 1). For datasets spanning multiple decades, we attributed values to the decade most represented by the data. For instance, the failure rate for drug candidates tested between 1967 and 1970 was assigned to the 1960s, as three of the four analyzed years fall within that decade (Supplementary Table S3). While not perfect, this analysis provides a quick overview of clinical failure trends over the past six decades.

**Table 1:** Characteristics of the collected drug attrition data per decade (1960s – 2010s).

	1960s	1970s	1980s	1990s	2000s	2010s
No. drug attrition data points	14	14	8	5	11	8
Mean analyzed timespan (years)	5.6	4.4	6.3	10.4	7	3.3
Min. drug attrition (%)	77	70	68.8	81	86.2	78.3
Max. drug attrition (%)	98.2	89.3	84.8	97.6	95	94
Median drug attrition (%)	90.4	80.9	80.7	89	90	93
Mean drug attrition (%)	89.7	80.8	79.5	88.1	90.7	90.8

The mean length of the analyzed timespans ranges from 3.3 years (2010s) to 10.4 years (1990s) (Table 1). The mean clinical failure rate of drug candidates amounted to 89.7% in the 1960s, seemingly dropping in the following two decades to reach its lowest point of 79.5% during the 1980s. However, it increased again from the 1990s to the end of the 2010s, reaching 90.8% (approximately 91%) in the last decade (Fig. 2B, Table 1). Median drug attrition values display a high degree of similarity to the mean drug attrition values for all decades.

Notably, publications describing clinical failure rates in the 1960s, 70s, and 80s tend to examine fewer drug candidates, often under 100 and seldom more than 300. This may be due to the type of data sources used – mostly surveys, some of which later grew into databases like the CSDD database (Supplementary Table S3). In contrast, studies from the 1990s, 2000s, and 2010s generally evaluate much larger numbers, often exceeding 1,000 compounds. This increase likely reflects the growing availability of databases and information on the topic, as well as enhanced technical and computer-aided capabilities to screen vast numbers of compounds. Furthermore, while there are larger fluctuations in the attrition rates in the 1960s, 70s, 80s, and 90s, the values in the 2000s and 2010s show a greater similarity and fall between 87% and 95%, with the sole exception of 102 drug candidates investigated at AstraZeneca from 2012 to 2016 that show a mean attrition rate of 78.3% (Morgan et al., 2018).

Hence, with high certainty, the failure rate of drug candidates approximates 91% between 2010 and 2017 (the 2010s), showing no detectable improvement since the levels observed between 1963 and 1969 (the 1960s).

### 3.2 Data overlap

**Fig. 3:**
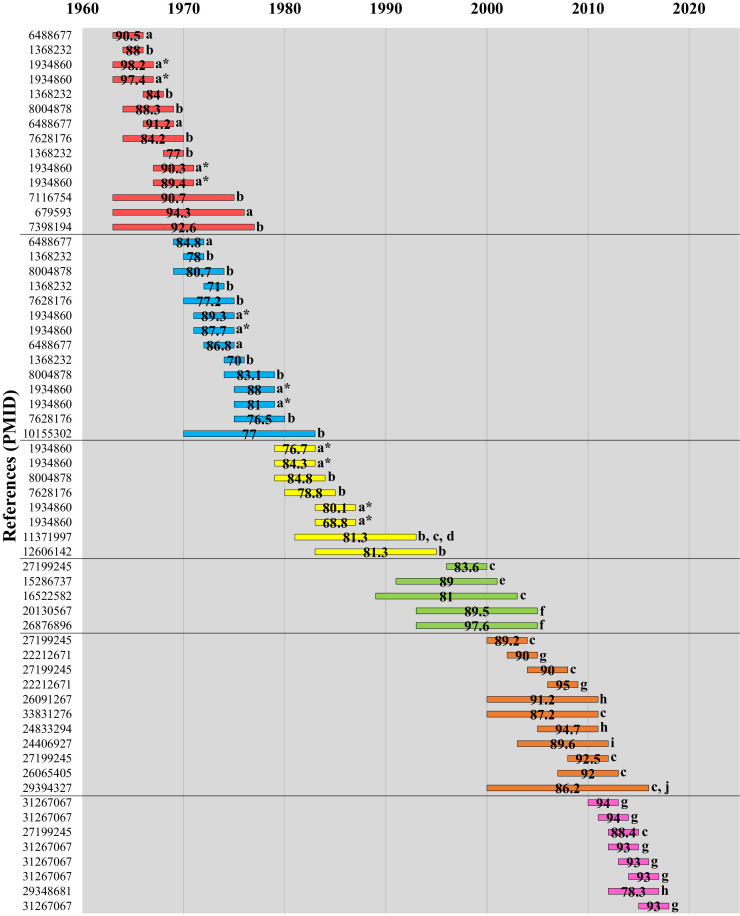
Overlap of data sets from individual drug attrition reports. Bars represent analysis periods from publications listed in Supplementary Table S3. Clinical failure rates (%) are shown inside bars. Bar colors correspond to decades in Fig. 2A and B. Letters (a-j) indicate data sources (detailed in Supplementary Table S3): a) Direct company surveys; b) CSDD database/surveys; c) Informa's Pharmaprojects; d) NDA Pipeline journal; e) Pharmaceutical Benchmarking Forum; f) IMS R&D Focus database; g) CMR International; h) Internal company data; i) Informa's BioMedTracker; j) Informa's Trialtrove Asterisks. (*) denote identical analysis periods for non-overlapping pharmaceutical companies (US vs. non-US) from (DiMasi et al., 1991).

The publications analyzed here do not disclose the names of the investigated drug candidates, thus, it is not possible to make an exact evaluation of the overlap between the individual studies and data sets. Nevertheless, to address the approximate degree of data overlap, we examined the analysis periods and data sources for each report (Fig. 3, Supplementary Table S3). As evident from Fig. 3, most of the individual drug attrition periods significantly overlap with adjacent studies in time, especially within the boundaries of each decade. While some studies exhibiting large temporal overlaps yield very similar results, in other cases there are evident discrepancies in such datasets. For example, Kola and Landis (2004) show a mean drug attrition of 89% between 1991 and 2000, whereas Adams and Brantner (2006) report a mean clinical drug attrition of 81% between 1989 and 2002 (Kola and Landis, 2004; Adams and Brantner, 2006). These differences are likely caused by the different data sources used in both studies – the Kola and Landis (2004) study focuses on data from the Pharmaceutical Benchmarking Forum (PBF) for drug candidates developed at 10 US and European firms, whereas Adams and Brantner (2006) use data from the Informa’s Pharmaprojects database and evaluates the attrition of 1055 drug candidates from the top 10 US pharmaceutical firms by sales.

A distinct evolution in data sources for drug attrition rate calculations is evident over time (Fig. 3). Analyses from the 1960s through early 1980s predominantly relied on author-conducted surveys of pharmaceutical companies, with the CSDD surveys later developing into an independent database. From the 1990s onward, drug attrition data increasingly originated from diverse databases. Informa's Pharmaprojects dominated in the 1990s and 2000s, while CMR International and Informa's BioMedTracker database became prominent in the 2010s. Although substantial overlap among these databases is likely, notable differences in drug failure rates for similar time periods, such as those reported by Kola and Landis (2004) and Adams and Brantner (2006), suggest some degree of variation between individual databases. This variability underscores the importance of considering multiple data sources when assessing long-term trends in drug attrition rates.

### 3.3 Biological reasons are the leading cause of drug attrition

To answer our second research question (RQ2), concerning the nature of the drug attrition reasons, we examined the data from 17 publications identified in our search that report reasons for drug failure in one or more phases of clinical trials (Supplementary Table S4). Wherever possible, the reasons for failure are addressed separately for each clinical phase, amounting to 36 individual data sets within the timespan 1963 - 2016. Like the analysis of the mean drug failure rates, there is an overlap in the data sources for the reasons for failure (Supplementary Table S4). However, as for RQ2 different clinical phases and combinations thereof are examined, we suspect that the overlap in the individual failed drug candidates is smaller than in the analysis for RQ1 above.

**Fig. 4:**
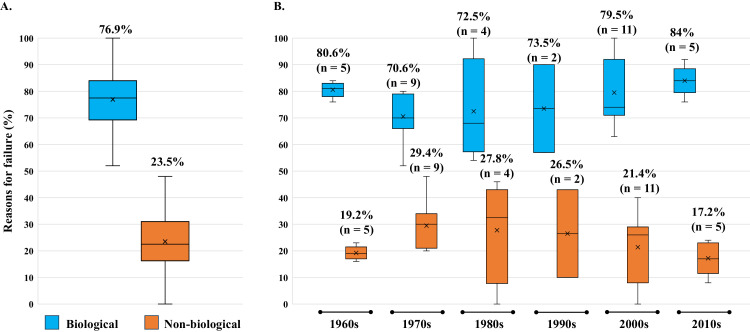
Reasons for failure. (A) Mean clinical failure rates of drug candidates attributed to biological (blue) and non-biological (orange) reasons, based on all data points (n = 36), calculated from Supplementary Table S4. (**B**) Decade-specific mean clinical failure rates due to biological (blue) and non-biological (orange) reasons from the 1960s to 2010s, calculated from Supplementary Table S4. The number of data points analyzed in each decade is indicated by 'n'. Numbers above each box represent the mean values. Boxes show the exclusive interquartile range (IQR) with median (line), mean (x), and minimum and maximum values (whiskers).

Because different parameters and endpoints are assessed during each phase of clinical trials, we expected that the reasons for failure would vary depending on the stage at which drug candidates fail. For example, since Phase I generally evaluates drug safety, we expected that most failures at this stage would be due to safety concerns. Nevertheless, the main purpose of our study was to analyze how much of the clinical failure can be approximately attributed to biological parameters that are usually evaluated in animals in preclinical studies and how much is due to commercial, strategic and other non-biological reasons. Therefore, we list the clinical causes of drug failure like safety concerns, insufficient efficacy or unfavorable pharmacokinetics and pharmacodynamics (PK/PD) in the category “biological reasons” and all other causes of failure in the category “non-biological reasons” (Fig. 4, Supplementary Table S4).

The sum of biological and non-biological reasons might slightly exceed 100%, most probably because multiple reasons can contribute to the failure of the same drug candidates. Interestingly, in all cases, the biological reasons are responsible for the majority of clinical failures irrespective of the failure stage. In 27 of the 36 datasets, they account for at least 70% of total failure. On average, 76.9% (or approximately 77%) of clinical drug failure can be attributed to biological reasons, mostly to efficacy and safety concerns, and 23.5% are due to commercial, strategic, and other non-biological reasons (Fig. 4A).

The ratio of biological to non-biological reasons for drug failure appears relatively stable across the six analyzed decades (Fig. 4B, Table 2). Approximately 70-80% of failures are attributed to biological factors, while 20-30% are due to non-biological factors in each decade. However, this analysis should be interpreted cautiously due to the high variability in the data sets and the differing predominance of clinical phases included in each decade.

**Table 2:** Characteristics of the collected data on reasons for drug attrition per decade (1960s – 2010s).

	1960s		1970s		1980s		1990s		2000s		2010s	
No. of datapoints	5		9		4		2		11		5	
Mean timespan (years)	3.8		6		4.8		3.5		7		3.2	
Reasons (Bio./Non-bio.)	Bio	Non	Bio	Non	Bio	Non	Bio	Non	Bio	Non	Bio	Non
Min values (%)	76	16	52	20	54	0	57	10	63	0	76	8
Max values (%)	84	23	80	48	100	46	90	43	100	40	92	24
Median values (%)	81	19	70	30	68	32.5	73.5	26.5	74	26	84	17
Mean values (%)	80.6	19.2	70.6	29.4	72.5	27.8	73.5	26.5	79.5	21.4	84.0	17.2

## 4 Discussion

In this evidence synthesis, we systematically analyzed trends in clinical drug failure rates and their underlying causes from 1963 onwards, using peer-reviewed literature indexed in PubMed that reported quantitative data on these outcomes. We identified 32 relevant publications, of which 25 reported 60 drug attrition rates (1963 – 2017), and 17 presented 36 datasets on reasons for clinical drug failure (1963 – 2016). Systematic analysis of the charted data reveals that the failure rate of drug candidates entering clinical trials during the 2000s and 2010s averages approximately 91%, comparable to levels observed in the 1960s. Biological factors emerged as the predominant cause of drug attrition across all stages of clinical trials, accounting for approximately 77% of failures on average. The main reported reasons included insufficient efficacy, safety concerns, and suboptimal pharmacokinetic/pharmacodynamic (PK/PD) profiles and bioavailability.

As detailed in the Limitations section, these estimates are subject to several constraints, including heterogeneity of data sources and methods, use of a single bibliographic database (PubMed), reliance on a single reviewer for title/abstract screening and incomplete access to some full texts. Nonetheless, the consistency of attrition levels and failure reason patterns across most decades and sources suggests that our synthesis provides a realistic approximation of long term trends in clinical drug development.

While multiple variables, including molecular modality and therapeutic area, undoubtedly influence an individual drug's probability of success, the prevalence of biological properties as the primary driver of overall drug failures suggests that current preclinical evaluation systems are not adequately predictive. In most development programmes, drug safety, efficacy and PK/PD properties must appear favourable in animal studies before first-in-human trials can commence. Yet our findings show that the majority of candidates still fail for biological reasons during clinical testing. This pattern is consistent with evidence that animal-to-human concordance is highly variable, both for specific toxicities and more generally when preclinical animal findings are compared with outcomes in clinical trials (Perel et al., 2007; Hackam and Redelmeier, 2006; Bailey et al., 2014, 2015).Well-known examples include non-steroidal anti-inflammatory drugs that are widely tolerated in humans but can cause severe renal or gastrointestinal toxicity in particular animal species, illustrating how species-specific biology can misrepresent human risk (Chalifoux et al., 2022). Such discordance reinforces the need for more human-relevant NAMs to assess safety and efficacy at clinically meaningful doses and endpoints.

### 4.1 NAMs addressing biological causes of drug attrition

Because a substantial share of clinical failures is safety related, NAMs that model human toxicity more accurately than standard animal tests are particularly relevant. For instance, in one study human liver-on-a-chip systems (Liver-Chips) have correctly predicted drug induced liver injury (DILI) for 13 out of 15 hepatotoxic drugs that had previously passed animal testing and entered clinical trials, while avoiding false positives among non-toxic substances (Ewart et al., 2021). Another similar study with human liver and cardiac organoids demonstrated the toxicity of 7 out of 10 toxic substances, all of which had previously evaded detection in animal tests (Skardal et al., 2020). These approaches directly target one of the principal biological failure modes highlighted in our analysis – unexpected clinical toxicity despite apparently acceptable nonclinical findings.

PK/PD-related problems are another major source of biologically driven attrition. Differences in species-specific physiology can lead to substantial dosing errors when human dose selection is extrapolated from animals. For example, large discrepancies in oral bioavailability between humans and animals like in the case of moxonidine (88% in humans vs. 5% in rats) can contribute to under- or over exposure in early clinical trials (He et al., 2003). Physiologically based pharmacokinetic (PBPK) modelling is now an established NAM to support human dose prediction and has provided pivotal evidence in many recent FDA and EMA approvals, particularly for drug–drug interactions, special populations (e.g. organ impairment, paediatrics) and oncology (Li et al., 2025; Paul et al., 2025; Grimstein et al., 2019). In parallel, AI-based expert systems trained exclusively on human clinical data like the IMPACT series by the German biotechnological company PharmaInformatic, can predict key PK parameters with accuracy that matches or exceeds traditional animal studies, enabling more reliable first in human dose estimation without additional animal data (Zietek et al., 2021). Such methods can effectively replace animal testing for pharmacokinetic assessment while enabling more accurate first-in-human dose selection and thereby potentially improving clinical trial success rates.

Efficacy-related failures the challenge of modelling human disease biology and treatment response. Advanced human based NAMs, such as organoids, 3D bioprinted tissues and organs on chips, offer the possibility of evaluating efficacy in systems that more closely recapitulate human pathophysiology than many animal-based models (van Berlo et al., 2021; Farhang Doost and Srivastava, 2024; Mallya et al., 2025). A notable case is a human-on-a-chip model for rare autoimmune neuropathies that provided efficacy data for an investigational new drug (IND) application, leading to Phase II clinical trial authorization (Rumsey et al., 2022). This example illustrates how such models can provide decision enabling evidence where species differences are particularly problematic.

Beyond these examples, thousands of NAMs are already catalogued for basic research and preclinical use, but their integration into late-stage drug development remains limited (BimmoH: Biomedical models Hub, 2026; NAT Database, 2020; NATworks, 2026). Recent work suggests that the main challenge is not the lack of translational assessment tools, but how to connect them into coherent, uncertainty-aware workflows that better support preclinical-to-Phase I decisions and improve translation success rates (Straccia et al., 2026).

### 4.2 Regulatory and policy momentum for reducing attrition

The regulatory environment is increasingly supportive of integrating NAMs into nonclinical assessment, with the explicit goal of improving the prediction of human safety and efficacy outcomes and thereby reducing late-stage attrition. The “FDA Modernization Act 2.0” from 2022 permits the use of NAMs in place of animal tests for safety and efficacy assessment in preclinical drug evaluation, and the FDA has since issued guidance and roadmaps to facilitate the validation and regulatory use of new methodologies (Rand, 2022; FDA, 2025). In parallel, the US National Institutes of Health (NIH) has launched an NIH-wide initiative to prioritize human-based research technologies and established the Office of Research Innovation, Validation, and Application (ORIVA) to coordinate the development and scale-up of non-animal approaches across its research portfolio (NIH, 2025). The NIH Common Fund’s Complement-ARIE programme is investing more than 100-150 million USD to develop, standardize and validate human-based NAMs, with the explicit aim of reducing reliance on animal models and improving clinical translation (NIH, 2026; Sunderic et al., 2025). In Europe, the European Commission’s roadmap towards phasing out animal testing for chemical safety assessments and the new ERA Action on “Accelerating NAMs to advance biomedical research and testing of medicinal products and medical devices” similarly seek to coordinate regulators, funders and industry around the development and uptake of NAMs in safety and efficacy testing ((Directorate-General for Internal Market, Industry, Entrepreneurship and SMEs (European Commission), 2024; Union PO of the E. Publications Office of the EU [Website], 2024; Roadmap for phasing out animal testing in chemical safety assessments: second workshop - European Commission, 2025; European Commission, 2026)). These initiatives are largely motivated by the need for improved clinical translation and more accurate prediction of safety and efficacy outcomes. Parallel to these regulatory developments, major pharmaceutical companies are increasingly investing in human‑based NAMs and publicly advocating their broader use in nonclinical safety assessment, underscoring growing industry confidence in these approaches (Beilmann et al., 2025). If NAMs are systematically implemented in late-stage preclinical drug evaluation, we might observe changes in the drug attrition rate in the coming decades.

### 4.3 Limitations and development needs of NAMs

Importantly, advanced human-based *in vitro* NAMs present both opportunities and challenges in drug development. can capture aspects of human tissue architecture and function that are absent from traditional cell lines, many still lack fully integrated immune components, vasculature and multi-organ interactions, and their longevity can limit the study of chronic effects. Increasing biological complexity can introduce variability related to donor characteristics and laboratory specific practices, complicating standardization and reproducibility. Moreover, complex organ-on-chip systems have historically been low throughput, although newer platforms that allow parallel operation of multiple chips and higher density assay formats are beginning to address this constraint (Goßmann et al., 2020; Lickiss et al., 2022; Emulate, Inc., 2025). Ongoing work on immunocompetent and vascularized models, adherence to good cell culture practices, standardized data reporting and formal model qualification will be essential to ensure that *in vitro* NAMs deployed in drug development are robust and reproducible (Nguyen et al., 2018; Homan et al., 2019; Huttala et al., 2019; Maurer et al., 2019; Wagar et al., 2021; Rogal et al., 2022; Salmon et al., 2022; Pamies et al., 2022). Increased investment in both development and validation of NAMs will be critical for their timely improvement and implementation in drug assessment protocols.

### 4.4 Animal-based models, standardization and attrition

Improving the predictive value of preclinical testing has long been pursued through greater standardization of animal studies, yet our analysis indicates that such efforts have not translated into lower overall clinical failure rates. Environmental standardization may paradoxically even reduce the reproducibility and external validity of animal experiments by minimizing biological variability in ways that do not reflect patient populations, contributing to inconsistent results across laboratories and disease models (Richter et al., 2009). Despite internationally harmonized safety guidelines, such as those issued by the International Council for Harmonisation of Technical Requirements for Pharmaceuticals for Human Use (ICH) since the 1990s, researchers and pharmaceutical companies continue to report low reproducibility of preclinical data, partly due to inadequate cell lines and animal data (Prinz et al., 2011; Begley and Ellis, 2012). The fact that therapeutic areas with particularly intensive animal use – such as oncology, neurology and cardiovascular disease – often exhibit some of the highest attrition rates further suggests that simply increasing the quantity or standardization of animal testing is unlikely to resolve the core problem (Thomas et al., 2021).

Our findings must also be interpreted in the context of various confounding factors, including market structure, economic developments, and legislative changes, that have influenced drug development over the past six decades. An analysis of FDA-approved drugs from 1950 to 2008 reveals a constant market entry rate despite exponentially increasing development costs (Munos, 2009). It highlights the limitations of established R&D models and a shift in innovation from large to small pharmaceutical companies, suggesting the need for novel strategies like open innovation to enhance productivity. Additionally, the pharmaceutical patent system has been criticized for potentially diminishing financial incentives for innovation (Grootendorst et al., 2011).

Legislation aimed at enhancing human participant protection and diversifying clinical trial participation may have contributed to increased drug attrition due to safety and efficacy concerns (FDA, 2018; European Commission, 2001; European Parliament, 2004). However, these regulatory changes likely improved the overall evaluation of drug candidates, primarily eliminating marginal compounds while preserving those with favorable biological properties. The persistently high drug failure rates in recent decades are thus more likely attributable to the poor translatability of current preclinical evaluations, which heavily rely on animal testing, in accurately predicting human biological responses.

### 4.5 Limitations

This study is primarily limited by the heterogeneity of the underlying reports, which differ in region, data source, time period, sample size (58–21,143 compounds for failure rates; 8–503 for failure reasons) and clinical phase coverage, with potential redundancy from overlapping datasets (Fig. 3). Earlier investigations (1960s-1980s) often rely on private surveys rather than official databases, raising concerns about reporting bias, while methodologies for calculating success/attrition rates and the distribution of molecular types, disease areas, and development settings also vary. Consequently, we did not perform a formal meta-analysis but instead synthesized findings narratively and with descriptive statistics at the decade level. Although these factors may influence the results to an undetermined extent, the concordance across most investigations within each decade suggests that our estimates realistically reflect long-term drug failure trends.

A further limitation is our exclusive use of PubMed for the systematic search, which may have missed relevant studies not indexed in this database and thereby introduced selection bias and constrained generalizability. PubMed was chosen for this analysis because of its comprehensive coverage of biomedical literature, wide use and focus on peer-reviewed studies, while resource constraints precluded inclusion of subscription-based databases such as Embase or Web of Science. As our aim was to map the breadth of evidence and major trends rather than to perform an exhaustive systematic review, we relied on detailed search strategies within PubMed, but acknowledge that future analyses of drug attrition would benefit from integrating additional databases to capture a broader set of studies and further validate our findings.

A further methodological limitation is that titles and abstracts were screened by a single reviewer before full text assessment, whereas full text screening and data extraction were performed independently by two reviewers. Relying on a single screener at the title/abstract stage may have increased the risk of inadvertently excluding some eligible studies, and this should be borne in mind when interpreting our findings.

Another limitation is that a subset of 109 PubMed records could not be included because no full text was available. Many were older records, and most had no link in PubMed and often no abstract, indicating that they were not readily accessible online.

Our findings are also constrained by the search terms used, which may have missed studies employing alternative terminology for drug attrition. In addition, we restricted inclusion to peer-reviewed, English-language research available online, which may have led to omission relevant non-English publications, pharmaceutical reports and non-peer-reviewed conference proceedings. Nevertheless, reports from the Biotechnology Innovation Organization (BIO), Biomedtracker and Amplion show comparable clinical attrition rates, increasing from 90.4% to 93.3% between 2005 and 2023 (Thomas et al., 2021, 2016; Chancellor, 2024). This alignment suggests that despite the language and publication type limitations, our analysis likely captures the broader trends in drug development attrition.

Another limitation of this study is the rapid evolution of drug development, potentially leading to relevant publications appearing after our search was conducted. For instance, a recent analysis of drug candidates from 18 leading pharmaceutical companies between 2006 and 2022 reported an average success rate of 14.3% (i.e. a failure rate of 85.7%) from Phase I, significantly higher than most studies in our analysis and the BIO-Biomedtracker-Amplion reports for a similar period (Schuhmacher et al., 2025). This discrepancy may be attributed to differences in methodologies, company selection, and inclusion criteria, such as the analysis of biologics.

This review is designed as a systematic analysis focused on quantifying clinical drug attrition rates and leading reasons for failure over the past six decades, not to resolve all underlying causal mechanisms. It does not examine psychological and organizational factors within companies (e.g. portfolio management, “early kill/fail fast” strategies, differing trust in animal studies vs. NAMs) or external contextual changes (regulation, trial standards, scientific advances, industry practices) that may independently shape attrition patterns (Paul et al., 2010). A more detailed analysis of how such decision-making cultures and specific regulatory, technological or market developments influence failure rates lies beyond our current scope but represents an important direction for future work building on these findings.

Potential for bias exists due to some authors’ affiliations with organizations advocating for or developing non-animal research methods and models. However, we minimized this risk through prospective protocol registration, predefined and consistently applied criteria, independent, blinded data extraction by two reviewers, and consensus-based oversight involving authors from academic, industry, and NGO backgrounds. We believe these measures substantially reduce the risk of bias in our findings.

## 5 Conclusions

In conclusion, this systematic evidence synthesis reveals that the overall clinical drug failure rate has remained persistently high – -approximately 91% over the past two decades (2000s-2010s) with little improvement since the 1960s. Notably, about 77% of failures are attributed to biological factors such as inadequate clinical safety and efficacy, which are parameters typically assessed in preclinical animal studies. These findings highlight ongoing challenges in translating preclinical results to successful clinical outcomes and underscore the need for continued evaluation and modernization of preclinical testing strategies.

Recent investments and regulatory changes are accelerating the development and acceptance of NAMs, supported by a growing body of evidence for their ability to enhance the predictivity and human relevance of preclinical drug testing. Realizing their full potential will require not only ongoing research and systematic integration into drug development pipelines, but also substantial shifts in funding priorities and broader acceptance within the scientific and regulatory communities.

Our work provides a comprehensive, longitudinal synthesis of drug attrition trends and reasons for failure, offering valuable context for stakeholders seeking to enhance drug development processes. As the field evolves, embracing innovative, human-relevant approaches, supported by recent scientific and regulatory initiatives, may help address longstanding challenges in preclinical testing. Continued, systematic monitoring of new methodologies and policy changes will be essential to identify strategies that genuinely improve the translation of preclinical findings into successful therapies for patients.

## Declaration of generative AI and AI-assisted technologies in the writing process

During the preparation of this work the authors used Perplexity AI in order to improve the readability of the manuscript. After using this tool, the authors reviewed and edited the content as needed and take full responsibility for the content of the published article.

## CRediT authorship contribution statement

**Dilyana Filipova:** Writing – review & editing, Writing – original draft, Visualization, Supervision, Project administration, Methodology, Investigation, Formal analysis, Data curation, Conceptualization. **Wolfgang Boomgaarden:** Writing – review & editing, Visualization, Methodology, Investigation, Formal analysis, Data curation. **Leyla Fox:** Writing – review & editing, Visualization, Investigation, Formal analysis, Data curation. **Andrew Knight:** Writing – review & editing, Investigation, Conceptualization. **Bettina Lickiss:** Writing – review & editing, Investigation, Conceptualization. **Christian Maass:** Writing – review & editing, Investigation, Conceptualization. **Gaby Neumann:** Writing – review & editing, Investigation, Conceptualization. **Justine Watkins:** Writing – review & editing, Investigation, Conceptualization. **Kathrin Herrmann:** Writing – review & editing, Investigation, Conceptualization. **Lorna Ewart:** Writing – review & editing, Investigation, Conceptualization. **Matthias Gossmann:** Writing – review & editing, Investigation, Conceptualization. **Merel Ritskes-Hoitinga:** Writing – review & editing, Investigation, Conceptualization. **Tamara Zietek:** Writing – review & editing, Writing – original draft, Visualization, Supervision, Project administration, Methodology, Investigation, Formal analysis, Data curation, Conceptualization.

## Declaration of competing interest

The authors declare the following financial interests/personal relationships which may be considered as potential competing interests:

Wolfgang Boomgaarden reports a relationship with PharmaInformatic that includes: employment. Bettina Lickiss reports a relationship with innoVitro GmbH that includes: employment. Christian Maass reports a relationship with esqLABS Gmbh that includes: employment. Lorna Ewart reports a relationship with Emulate Inc that includes: employment. Matthias Gossmann reports a relationship with innoVitro GmbH that includes: employment. Given her role as a member of the Editorial Board of the NAM Journal, Merel Ritskes-Hoitinga had no involvement in the peer review of this article and had no access to information regarding its peer review. Full responsibility for the editorial process for this article was delegated to another journal editor. If there are other authors, they declare that they have no known competing financial interests or personal relationships that could have appeared to influence the work reported in this paper.

## Data Availability

All data supporting the findings of this study are included within the manuscript and its supplementary information.

## Appendix Supplementary materials

Image, application 1

Image, application 2

Image, application 3

Image, application 4

Image, application 5
