# Supplementary material for: Need for NAMs: A systematic evidence synthesis revealing over half a century of drug development failure
Source: NAM J. 2026 Jul 21;2:100119. doi: 10.1016/j.namjnl.2026.100119 (PMC13453574; doi:10.1016/j.namjnl.2026.100119)
Supplement: Supplementary file 3 [file mmc3.pdf]

Supplementary Table S3. Clinical failure rates in drug development.

<u>Note</u>: Abbreviations: NCE – new chemical entity; IND – investigational new drug; NDA – new drug application.

Failure rate (%)	Analysis period	Decade	Data set and source	PMID	Reference
90.5	1963-1965	1960s	211 NCEs from 39 US-based companies, accounting for 90% of NCEs investigated by US firms	6488677	(1)
88	1964-1965	1960s	224 NCEs; data from two CSDD surveys of the US pharmaceutical industry	1368232	(2)
98.2	1963-1966	1960s	333 NCEs from 31 US-owned firms; data from two surveys	1934860	(3)
97.4	1963-1966	1960s	78 NCEs from 10 non-US-owned firms; data from two surveys	1934860	(3)
84	1966-1967	1960s	178 NCEs; data from two CSDD surveys of the US pharmaceutical industry	1368232	(2)
88.3	1964-1968	1960s	ca. 294 NCEs (1,177 for 1964-1981) at 22 US-owned firms and 14 US subsidiaries of foreign firms; data from the CSDD triennial survey of firms operating in the US and a separate CSDD annual survey of firms that had received an NDA approval	8004878	(4)
91.2	1966-1968	1960s	136 NCEs from 39 US-based companies, accounting for 90% of NCEs investigated by US firms	6488677	(1)
84.2	1964-1969	1960s	576 NCEs from US firms; data from the Tufts CSDD database	7628176	(5)
77	1968-1969	1960s	185 NCEs; data from two CSDD surveys of the US pharmaceutical industry	1368232	(2)
90.3	1967-1970	1960s	247 NCEs from US-owned firms; data from two surveys of 31 US-owned firms	1934860	(3)
89.4	1967-1970	1960s	94 NCEs from non-US-owned firms; data from two surveys of 10 US subsidiaries of foreign firms	1934860	(3)
90.7	1963-1979	1960s	1040 NCEs from 39 US owned companies; data from a 1980 CSDD survey	7116754	(6)
94.3	1963-1975	1960s	1,029 NCEs with INDs, of which 59 NDA approvals; data from a survey of all Pharmaceutical Manufacturers Association (PMA) members and generic drug suppliers in the US and firms likely to be involved in drug development; data from 46 firms, estimated to account for 95% of US-owned NCEs and nearly all foreign-owned NCEs	679593	(7)
92.6	1963-1976	1960s	794 NCEs from 39 US-firms; data from the 1977 CSDD survey	7398194	(8)
84.8	1969-1971	1970s	139 NCEs from 39 US-based companies, accounting for 90% of NCEs investigated by US firms	6488677	(1)
78	1970-1971	1970s	161 NCEs; data from two CSDD surveys of the US pharmaceutical industry	1368232	(2)
80.7	1969-1973	1970s	ca. 294 NCEs (1,177 for 1964-1981) at 22 US-owned firms and 14 US subsidiaries of foreign firms; data from the CSDD triennial survey of firms operating in the US and a separate CSDD annual survey of firms that had received an NDA approval	8004878	(4)
71	1972-1973	1970s	145 NCEs; data from two CSDD surveys of the US pharmaceutical industry	1368232	(2)
77.2	1970-1974	1970s	378 NCEs from US firms; data from the Tufts CSDD database	7628176	(5)
89.3	1971-1974	1970s	251 NCEs from US-owned firms; data from two surveys of 31 US-owned firms	1934860	(3)
87.7	1971-1974	1970s	65 NCEs from non-US-owned firms; data from two surveys of 10 US subsidiaries of foreign firms	1934860	(3)
86.8	1972-1974	1970s	91 NCEs from 39 US-based companies, accounting for 90% of NCEs investigated by US firms	6488677	(1)
70	1974-1975	1970s	132 NCEs; data from two CSDD surveys of the US pharmaceutical industry	1368232	(2)

83.1	1974-1978	1970s	ca. 294 NCEs (1,177 for 1964-1981) at 22 US-owned firms and 14 US subsidiaries of foreign firms; data from the CSDD triennial survey of firms operating in the US and a separate CSDD annual survey of firms that had received an NDA approval	8004878	(4)
88	1975-1978	1970s	173 NCEs from US-owned firms; data from two surveys of 31 US-owned firms	1934860	(3)
81	1975-1978	1970s	58 NCEs from non-US-owned firms; data from two surveys of 10 US subsidiaries of foreign firms	1934860	(3)
76.5	1975-1979	1970s	247 NCEs from US firms; data from the Tufts CSDD database	7628176	(5)
77	1970-1982	1970s	93 NCEs from 12 US-owned pharmaceutical firms; data from the Tufts CSDD database	10155302	(9)
76.7	1979-1982	1980s	201 NCEs from US-owned firms; data from two surveys of 31 US-owned firms	1934860	(3)
84.3	1979-8192	1980s	83 NCEs from non-US-owned firms; data from two surveys of 10 US subsidiaries of foreign firms	1934860	(3)
84.8	1979-1983	1980s	ca. 294 NCEs (1,177 for 1964-1981) at 22 US-owned firms and 14 US subsidiaries of foreign firms; data from the CSDD triennial survey of firms operating in the US and a separate CSDD annual survey of firms that had received an NDA approval	8004878	(4)
78.8	1980-1984	1980s	326 NCEs from US firms; data from the Tufts CSDD database	7628176	(5)
80.1	1983-1986	1980s	201 NCEs from US-owned firms; data from two surveys of 31 US-owned firms	1934860	(3)
68.8	1983-1986	1980s	64 NCEs from non-US-owned firms; data from two surveys of 10 US subsidiaries of foreign firms	1934860	(3)
81.3	1981-1992	1980s	460 self-originated NCEs from US-owned companies; data from the Tufts CSDD database and two public sources: Informa's Pharmaprojects and the NDA pipeline	11371997	(10)
81.3	1983-1994	1980s	486 NCEs with final decision; data from the Tufts CSDD database	12606142	(11)
83.6	1996-1999	1990s	2603 drugs; data from Informa's Pharmaprojects database	27199245	(12)
89	1991-2000	1990s	compounds at top 10 firms in the US and Europe; data from Datamonitor in the Pharmaceutical Benchmarking Forum (PBF)	15286737	(13)
81	1989-2002	1990s	1055 compounds from top 10 US firms by drug count; data from Informa's Pharmaprojects database	16522582	(14)
89.5	1993-2004	1990s	1316 NCEs from top 50 firms by sales; data from the IMS R&D Focus investigational drug pipeline database	20130567	(15)
97.6	1993-2004	1990s	2562 drugs by 406 US firms; data from the IMS Health's R&D Focus database	26876896	(16)
89.2	2000-2003	2000s	2965 drugs; data from Informa's Pharmaprojects database	27199245	(12)
90	2002-2004	2000s	drugs at 14 firms; data from CMR International	22212671	(17)
90	2004-2007	2000s	3776 drugs; data from Informa's Pharmaprojects database	27199245	(12)
95	2006-2008	2000s	drugs at 14 firms; data from CMR International	22212671	(17)
91.2	2000-2010	2000s	379 small-molecule drug candidates; data from AstraZeneca, Eli Lilly and Company, GlaxoSmithKline and Pfizer (directly provided)	26091267	(18)
87.2	2000-2010	2000s	3,999 drugs; data from Informa's Pharmaprojects database	33831276	(19)
94.7	2005-2010	2000s	142 drugs at AstraZeneca	24833294	(20)
89.6	2003-2011	2000s	4451 drugs from 835 companies; data from Informa's BioMedTracker database	24406927	(21)
92.5	2008-2011	2000s	5144 drugs; data from Informa's Pharmaprojects database	27199245	(12)
92	2007-2012	2000s	all novel compounds from the Informa's Pharmaprojects database	26065405	(22)
86.2	2000-2015	2000s	21,143 compounds; Citeline data provided by Informa Pharma Intelligence, combining individual clinical trial information from Trialtrove and drug approval data from Pharmaprojects	29394327	(23)
94	2010-2012	2010s	compounds from 30 firms; data from the CMR International consortium, including data from ca. 30 large mid-sized and small firms, collectively representing ~80% of the top 20 biopharmaceutical companies	31267067	(24)

94	2011-2013	2010s	compounds from 30 firms; data from the CMR International consortium, including data from ca. 30 large mid-sized and small firms, collectively representing ~80% of the top 20 biopharmaceutical companies	31267067	(24)
88.4	2000-2003	2010s	4808 drugs; data from Informa's Pharmaprojects database	27199245	(12)
93	2012-2014	2010s	compounds from 30 firms; data from the CMR International consortium, including data from ca. 30 large mid-sized and small firms, collectively representing ~80% of the top 20 biopharmaceutical companies	31267067	(24)
93	2013-2015	2010s	compounds from 30 firms; data from the CMR International consortium, including data from ca. 30 large mid-sized and small firms, collectively representing ~80% of the top 20 biopharmaceutical companies	31267067	(24)
93	2014-2016	2010s	compounds from 30 firms; data from the CMR International consortium, including data from ca. 30 large mid-sized and small firms, collectively representing ~80% of the top 20 biopharmaceutical companies	31267067	(24)
78.3	2012-2016	2010s	102 drugs at AstraZeneca	29348681	(25)
93	2015-2017	2010s	compounds from 30 firms; data from the CMR International consortium, including data from ca. 30 large mid-sized and small firms, collectively representing ~80% of the top 20 biopharmaceutical companies	31267067	(24)

Supplementary Table S3 References:

1. Sheck L, Cox C, Davis HT, Trimble AG, Wardell WM, Hansen R. Success rates in the United States drug development system. Clin Pharmacol Ther. 1984;36(5):574–83.

2. Bienz-Tadmor B, DiCerbo PA, Tadmor G, Lasagna L. Biopharmaceuticals and Conventional Drugs: Clinical Success Rates. Bio/Technology. 1992 May;10(5):521–5.

3. DiMasi JA, Bryant NR, Lasagna L. New drug development in the United States from 1963 to 1990. Clin Pharmacol Ther. 1991;50(5–1):471–86.

4. DiMasi JA, Seibring MA, Lasagna L. New drug development in the United States from 1963 to 1992. Clin Pharmacol Ther. 1994;55(6):609–22.

5. DiMasi JA. Success rates for new drugs entering clinical testing in the United States. Clin Pharmacol Ther. 1995;58(1):1–14.

6. Wardell WM, May MS, Trimble AG. New drug development by United States pharmaceutical firms: With analyses of trends in the acquisition and origin of drug candidates, 1963–1979*. Clin Pharmacol Ther. 1982;32(4):407–17.

7. Wardell WM, Hassar M, Anavekar SN. The rate of development of new drugs in the United States, 1963 through 1975. Clin Pharmacol Ther. 1978;24(2):133–45.

8. Wardell WM, DiRaddo J, Gene Trimble A. Development of new drugs originated and acquired by United States–owned pharmaceutical firms, 1963–1976. Clin Pharmacol Ther. 1980;28(2):270–7.

9. DiMasi JA, Hansen RW, Grabowski HG, Lasagna L. Research and development costs for new drugs by therapeutic category. A study of the US pharmaceutical industry. PharmacoEconomics. 1995 Feb;7(2):152–69.

10. DiMasi JA. Risks in new drug development: Approval success rates for investigational drugs. Clin Pharmacol Ther. 2001;69(5):297–307.

11. DiMasi JA, Hansen RW, Grabowski HG. The price of innovation: new estimates of drug development costs. J Health Econ. 2003 Mar 1;22(2):151–85.

12. Smietana K, Siatkowski M, Møller M. Trends in clinical success rates. Nat Rev Drug Discov. 2016 Jun 1;15(6):379–80.

13. Kola I, Landis J. Can the pharmaceutical industry reduce attrition rates? Nat Rev Drug Discov. 2004 Aug;3(8):711–6.

14. Adams CP, Brantner VV. Estimating The Cost Of New Drug Development: Is It Really $802 Million? Health Aff (Millwood). 2006 Mar;25(2):420–8.

15. DiMasi JA, Feldman L, Seckler A, Wilson A. Trends in Risks Associated With New Drug Development: Success Rates for Investigational Drugs. Clin Pharmacol Ther. 2010;87(3):272–7.

16. Dahlin E, Nelson GM, Haynes M, Sargeant F. Success rates for product development strategies in new drug development. J Clin Pharm Ther. 2016;41(2):198–202.

17. Arrowsmith J. A decade of change. Nat Rev Drug Discov. 2012 Jan 1;11(1):17–8.

18. Waring MJ, Arrowsmith J, Leach AR, Leeson PD, Mandrell S, Owen RM, et al. An analysis of the attrition of drug candidates from four major pharmaceutical companies. Nat Rev Drug Discov. 2015 Jul;14(7):475–86.

19. Yamaguchi S, Kaneko M, Narukawa M. Approval success rates of drug candidates based on target, action, modality, application, and their combinations. Clin Transl Sci. 2021;14(3):1113–22.

20. Cook D, Brown D, Alexander R, March R, Morgan P, Satterthwaite G, et al. Lessons learned from the fate of AstraZeneca's drug pipeline: a five-dimensional framework. Nat Rev Drug Discov. 2014 Jun;13(6):419–31.

21. Hay M, Thomas DW, Craighead JL, Economides C, Rosenthal J. Clinical development success rates for investigational drugs. Nat Biotechnol. 2014 Jan;32(1):40–51.

22. Smietana K, Ekstrom L, Jeffery B, Møller M. Improving R&D productivity. Nat Rev Drug Discov. 2015 Jul 1;14(7):455–6.

23. Wong CH, Siah KW, Lo AW. Estimation of clinical trial success rates and related parameters. Biostatistics. 2019 Apr 1;20(2):273–86.

24. Dowden H, Munro J. Trends in clinical success rates and therapeutic focus. Nat Rev Drug Discov. 2019 May 8;18(7):495–6.

25. Morgan P, Brown DG, Lennard S, Anderton MJ, Barrett JC, Eriksson U, et al. Impact of a five-dimensional framework on R&D productivity at AstraZeneca. Nat Rev Drug Discov. 2018 Mar;17(3):167–81.
