# Supplementary material for: Need for NAMs: A systematic evidence synthesis revealing over half a century of drug development failure
Source: NAM J. 2026 Jul 21;2:100119. doi: 10.1016/j.namjnl.2026.100119 (PMC13453574; doi:10.1016/j.namjnl.2026.100119)
Supplement: Supplementary file 4 [file mmc4.pdf]

Supplementary Table S4. Reasons for clinical failure.

PMID	Reasons for failure (%)*		Failure at stage(s)	Time period	Decade	Elaboration / Data source	Reference
	Biological	Non-biological					
7116754	81	19	Phase I-NDA	1963-1969	1960s	Failures at 39 US firms<br><br>Biological: Scientific reasons: 81%<br>Non-biolofical: Economic reasons: 15%; Other reasons: 4%<br><br>Data source: CSDD 1980 survey of 39 US-owned firms	(1)
7398194	68	32	Phase I-NDA	1963-1976	1970s	503 failed self-originated NCEs from 39 US-owned firms<br><br>Biological: Scientific reasons: 68%<br>Non-biolofical: Other reasons: 32%<br><br>Data source: CSDD 1977 survey of 39 US-owned firms	(2)
7398194	52	48	Phase I-NDA	1963-1976	1970s	98 failed acquired NCEs from 39 US-owned firms<br><br>Biological: Scientific reasons: 52%<br>Non-biolofical: Other reasons: 48%<br><br>Data source: CSDD 1977 survey of 39 US-owned firms	(2)
1368232	84	16	Phase I-NDA	1964-1965	1960s	224 NCEs in total, of which 88% failed<br><br>Biological: Scientific reasons: 84%<br>Non-biological: unspecified<br><br>Data source: CSDD surveys	(3)
7628176	76	23	Phase I-NDA	1964-1969	1960s	406 failed NCEs from US firms<br><br>Biological: Safety: 23%; Efficacy: 53%<br>Non-biological: Economics: 18%; Unclassified: 5%<br><br>Data source: Tufts CSDD database	(4)
1368232	82	18	Phase I-NDA	1966-1967	1960s	178 NCEs in total, of which 84% failed	(3)

						Biological: Scientific reasons: 82%<br>Non-biological: unspecified<br><br>Data source: CSDD surveys	
1368232	80	20	Phase I-NDA	1968-1969	1960s	185 NCEs in total, of which 77% failed<br><br>Biological: Scientific reasons: 80%<br>Non-biological: unspecified<br><br>Data source: CSDD surveys	(3)
1368232	80	20	Phase I-NDA	1970-1971	1970s	161 NCEs in total, of which 78% failed<br><br>Biological: Scientific reasons: 80%<br>Non-biological: unspecified<br><br>Data source: CSDD surveys	(3)
7116754	70	30	Phase I-NDA	1970-1974	1970s	Failures at 39 US firms<br><br>Biological: Scientific reasons: 70%<br>Non-biolofical: Economic reasons: 16%; Other reasons: 14%<br><br>Data source: CSDD 1980 survey of 39 US-owned firms	(1)
7628176	74	26	Phase I-NDA	1970-1974	1970s	247 failed NCEs from US firms<br><br>Biological: Safety: 28%; Efficacy: 46%<br>Non-biological: Economics: 22%; Unclassified: 4%<br><br>Data source: Tufts CSDD database	(4)
1368232	79	21	Phase I-NDA	1972-1973	1970s	145 NCEs in total, of which 71% failed<br><br>Biological: Scientific reasons: 79%<br>Non-biological: unspecified<br><br>Data source: CSDD surveys	(3)
1368232	79	21	Phase I-NDA	1974-1975	1970s	132 NCEs in total, of which 70% failed<br><br>Biological: Scientific reasons: 79%<br>Non-biological: unspecified<br><br>Data source: CSDD surveys	(3)

7116754	64	36	Phase I-NDA	1975-1979	1970s	Failures at 39 US firms<br><br>Biological: Scientific reasons: 64%<br>Non-biolofical: Economic reasons: 26%; Other reasons: 10%<br><br>Data source: CSDD 1980 survey of 39 US-owned firms	(1)
7628176	69	31	Phase I-NDA	1975-1979	1970s	152 failed NCEs from US firms<br><br>Biological: Safety: 26%; Efficacy: 43%<br>Non-biological: Economics: 26%; Unclassified: 5%<br><br>Data source: Tufts CSDD database	(4)
7628176	69	31	Phase I-NDA	1980-1984	1980s	194 failed NCEs from US firms<br><br>Biological: Safety: 33%; Efficacy: 36%<br>Non-biological: Economics: 27%; Unclassified: 4%<br><br>Data source: Tufts CSDD database	(4)
11371997	54	46	Phase I-NDA	1981-1986	1980s	215 NCEs<br><br>Biological: Safety: 21.4%; Efficacy: 33%<br>Non-biological: Economics: 29.8%; Other: 15.8%<br><br>Data source: Tufts CSDD database, Informa’s Pharmaprojects and the NDA pipeline	(5)
7628176	67	34	Phase I-NDA	1985-1989	1980s	100 failed NCEs from US firms<br><br>Biological: Safety: 26%; Efficacy: 41%<br>Non-biological: Economics: 29%; Unclassified: 5%<br><br>Data source: Tufts CSDD database	(4)
1998766	100	0	product license application (equivalent to NDA submission)	1987-1989	1980s	71 unsuccessful product license application in the UK<br><br>Biological: Safety and Efficacy: 55/71; Safety only: 14/71; Efficacy only: 2/71<br><br>Data source: UK Committee on Safety of Medicines	(6)

11371997	57	43	Phase I-NDA	1987-1992	1990s	133 NCEs<br><br>Biological: Safety: 19.6%; Efficacy: 37.6%<br>Non-biological: Economics: 33.8%; Other: 9%<br><br>Data source: Tufts CSDD database, Informa's Pharmaprojects and the NDA pipeline	(5)
15286737	90	10	Phase I-NDA	1993	1990s	Failures at 10 pharmaceutical companies<br><br>Biological: Clinical safety: 10%; Efficacy: 30%; PK/bioavailability: 40%; Toxicology: 10%;<br>Non-biological: Commercial: 5%; Unknown: 5%<br><br>Data source: Datamonitor in the Pharmaceutical Benchmarking Forum (PBF)	(7)
27723879	73,9	26,2	Phase III	1998-2008	2000s	344 clinical failures<br><br>Biological: Efficacy: 56.7%; Safety: 17.2%<br>Non-biological: Commercial: 21.5%; Unknown: 4.7%<br><br>Data source: Informa's Pharmaprojects and Springer´s AdisInsight databases	(8)
15286737	70	40	Phase I-NDA	2000	2000s	Failures at 10 pharmaceutical companies<br><br>Biological: Clinical safety: 12%; Efficacy: 28%; PK/bioavailability: 10%; Toxicology: 20%;<br>Non-biological: Commercial: 20%; Formulation: 5%; Cost of goods: 10%; Unknown: 5%<br><br>Data source: Datamonitor in the Pharmaceutical Benchmarking Forum (PBF)	(7)
26091267	73	27	Phase I	2000-2010	2000s	157 failed compounds<br><br>Biological: Safety: 25%; PK/bioavailability: 16%; Toxicology: 13%; Scientific: 10%; Efficacy: 9%<br>Non-biological: Portfolio: 18%; Commercial: 6%; Formulation: 0.6%; Regulatory: 0.6%; Technical: 0.6%<br><br>Data source: Survey of four pharmaceutical firms	(9)

26091267	74	26	Phase II	2000-2010	2000s	89 failed compounds<br><br>Biological: Safety: 25%; PK/bioavailability: 1%; Toxicology: 8%; Scientific: 6%; Efficacy: 35%<br>Non-biological: Portfolio: 21%; Commercial: 4%<br><br>Data source: Survey of four pharmaceutical firms	(9)
24406927	63	37	Phase III	2003-2011	2000s	359 failed compounds<br><br>Biological: Efficacy: 54%; Safety: 9%<br>Non-biological: Commercial: 18%; Unknown: 19%<br><br>Data source: The BioMedTracker database	(10)
24406927	79	21	NDA submission	2003-2011	2000s	95 failed compounds<br><br>Biological: Efficacy: 48%; Safety: 31%<br>Non-biological: Commercial: 8%; Unknown: 13%<br><br>Data source: The BioMedTracker database	(10)
24833294	92	8	Phase I	2005-2010	2000s	27 failed drug projects<br><br>Biological: Safety: 62%; Efficacy: 15%; PK/PD: 15%<br>Non-biological: Strategy: 8%<br><br>Data source: AstraZeneca	(11)
24833294	92	8	Phase IIa	2005-2010	2000s	26 failed drug projects<br><br>Biological: Safety: 35%; Efficacy: 57%;<br>Non-biological: Strategy: 8%<br><br>Data source: AstraZeneca	(11)
24833294	100	0	Phase IIb	2005-2010	2000s	8 failed drug projects<br><br>Biological: Safety: 12%; Efficacy: 88%;<br><br>Data source: AstraZeneca	(11)
21283095	87	13	Phase III - NDA submission	2007-2010	2000s	83 Phase III and submission failures<br><br>Biological: Efficacy: 66%; Safety: 21%;<br>Non-biological: Commercial: 7%; Not disclosed: 6%<br><br>Data source: CMR International, Thomson	(12)

						Reuters Life Science Consulting	
21532551	71	29	Phase II	2008-2010	2000s	87 Phase II failures<br><br>Biological: Efficacy: 51%; Safety: 19%; PK/PD: 1%<br>Non-biological: Strategic: 29%<br><br>Data source: CMR International, Thomson Reuters Life Science Consulting	(13)
23903212	84	17	Phases II-NDA	2011-2012	2010s	105 clinical failures<br><br>Biological: Efficacy: 56%; Safety: 28%;<br>Non-biological: Strategic: 7%; Commercial: 5%; Operational: 5%<br><br>Data source: Thomson Reuters Life Science Consulting analysis of the journal Drugs of Today	(14)
29348681	85	15	Phase I	2012-2016	2010s	13 failed drug projects<br><br>Biological: Safety: 38,5%; Efficacy: 38,5%; PK/PD: 8%<br>Non-biological: Strategy: 15%<br><br>Data source: AstraZeneca	(15)
29348681	92	8	Phase II	2012-2016	2010s	13 failed drug projects<br><br>Biological: Safety: 8%; Efficacy: 84%<br>Non-biological: Strategy: 8%<br><br>Data source: AstraZeneca	(15)
27811931	76	24	Phases II-III	2013-2015	2010s	174 clinical failures<br><br>Biological: Efficacy: 52%; Safety: 24%<br>Non-biological: Commercial: 6%; Strategy: 15%; Operational: 3%<br><br>Data source: Thomson Reuters Life Science Consulting analysis of the journal Drugs of Today	(16)
28387389	83	22	Phases I-III	2016	2010s	59 failed drugs<br><br>Biological: Lack of efficacy: 63% (37/59); Adverse events: 10% (6/59); Unfavorable benefit-risk profile: 7% (4/59); (Negative) study results: 3% (2/59)<br>Non-biological: Strategic decision: 15% (9/59); High	(17)

						cost: 2% (1/59); Other: 5% (3/59)<br><br>Data source: Thomson Reuters Drug News and Integrity from Clarivate Analytics	

Supplementary Table S4 References:

1. Wardell WM, May MS, Trimble AG. New drug development by United States pharmaceutical firms: With analyses of trends in the acquisition and origin of drug candidates, 1963–1979*. Clin Pharmacol Ther. 1982;32(4):407–17.

2. Wardell WM, DiRaddo J, Gene Trimble A. Development of new drugs originated and acquired by United States–owned pharmaceutical firms, 1963–1976. Clin Pharmacol Ther. 1980;28(2):270–7.

3. Bienz-Tadmor B, DiCerbo PA, Tadmor G, Lasagna L. Biopharmaceuticals and Conventional Drugs: Clinical Success Rates. Bio/Technology. 1992 May;10(5):521–5.

4. DiMasi JA. Success rates for new drugs entering clinical testing in the United States. Clin Pharmacol Ther. 1995;58(1):1–14.

5. DiMasi JA. Risks in new drug development: Approval success rates for investigational drugs. Clin Pharmacol Ther. 2001;69(5):297–307.

6. Rawlins MD, Jefferys DB. Study of United Kingdom product licence applications containing new active substances, 1987-9. Br Med J. 1991 Jan 26;302(6770):223–5.

7. Kola I, Landis J. Can the pharmaceutical industry reduce attrition rates? Nat Rev Drug Discov. 2004 Aug;3(8):711–6.

8. Hwang TJ, Carpenter D, Lauffenburger JC, Wang B, Franklin JM, Kesselheim AS. Failure of Investigational Drugs in Late-Stage Clinical Development and Publication of Trial Results. JAMA Intern Med. 2016 Dec 1;176(12):1826.

9. Waring MJ, Arrowsmith J, Leach AR, Leeson PD, Mandrell S, Owen RM, et al. An analysis of the attrition of drug candidates from four major pharmaceutical companies. Nat Rev Drug Discov. 2015 Jul;14(7):475–86.

10. Hay M, Thomas DW, Craighead JL, Economides C, Rosenthal J. Clinical development success rates for investigational drugs. Nat Biotechnol. 2014 Jan;32(1):40–51.

11. Cook D, Brown D, Alexander R, March R, Morgan P, Satterthwaite G, et al. Lessons learned from the fate of AstraZeneca's drug pipeline: a five-dimensional framework. Nat Rev Drug Discov. 2014 Jun;13(6):419–31.

12. Arrowsmith J. Phase III and submission failures: 2007–2010. Nat Rev Drug Discov. 2011 Feb 1;10(2):87–87.

13. Arrowsmith J. Phase II failures: 2008–2010. Nat Rev Drug Discov. 2011 May 1;10(5):328–9.

14. Arrowsmith J, Miller P. Phase II and Phase III attrition rates 2011–2012. Nat Rev Drug Discov. 2013 Aug 1;12(8):569–569.

15. Morgan P, Brown DG, Lennard S, Anderton MJ, Barrett JC, Eriksson U, et al. Impact of a five-dimensional framework on R&D productivity at AstraZeneca. Nat Rev Drug Discov. 2018 Mar;17(3):167–81.

16. Harrison RK. Phase II and phase III failures: 2013–2015. Nat Rev Drug Discov. 2016 Dec 1;15(12):817–8.

17. Graul AI, Dulsat C, Tracy M, Cruces E. The year's new drugs & biologics 2016: Part II - Trends and highlights of an unforgettable year. Drugs Today Barc Spain 1998. 2017 Feb;53(2):117–58.
