# Supplementary material for: Need for NAMs: A systematic evidence synthesis revealing over half a century of drug development failure
Source: NAM J. 2026 Jul 21;2:100119. doi: 10.1016/j.namjnl.2026.100119 (PMC13453574; doi:10.1016/j.namjnl.2026.100119)
Supplement: Supplementary file 5 [file mmc5.pdf]

Supplementary Table S5. Quality assessment checklist.

The presence of each data items increased the overall quality score of the analyzed studies by one point, with a maximal quality score of 8 points. The publications are grouped based on the presence of data on both drug attrition rates and reasons for failure, only attrition rates, or only reasons for failure.

Extracted data	PMID	Citation	Attrition rates present?	Reasons for failure present?	Data are original?	The analysis period is defined?	The analysis sample is defined?	The number of drug candidates is disclosed?	The number of pharmaceutical companies is disclosed?	The type of drug candidates is disclosed?	**Reporting score**
Attrition rates and reasons for failure	7116754	(1)	1	1	1	1	1	1	1	1	**8**
	7398194	(2)	1	1	1	1	1	1	1	1	**8**
	7628176	(3)	1	1	1	1	1	1	1	1	**8**
	11371997	(4)	1	1	1	1	1	1	1	1	**8**
	24406927	(5)	1	1	1	1	1	1	1	1	**8**
	24833294	(6)	1	1	1	1	1	1	1	1	**8**
	29348681	(7)	1	1	1	1	1	1	1	1	**8**
	1368232	(8)	1	1	1	1	1	1	0	1	**7**
	26091267	(9)	1	1	1	1	1	1	1	0	**7**
	15286737	(10)	1	1	1	1	1	0	1	0	**6**
Attrition rates	679593	(11)	1	0	1	1	1	1	1	1	**7**
	1934860	(12)	1	0	1	1	1	1	1	1	**7**
	8004878	(13)	1	0	1	1	1	1	1	1	**7**
	10155302	(14)	1	0	1	1	1	1	1	1	**7**
	16522582	(15)	1	0	1	1	1	1	1	1	**7**
	20130567	(16)	1	0	1	1	1	1	1	1	**7**
	6488677	(17)	1	0	1	1	1	1	0	1	**6**
	12606142	(18)	1	0	1	1	1	1	0	1	**6**
	26876896	(19)	1	0	1	1	1	1	1	0	**6**
	27199245	(20)	1	0	1	1	1	1	0	1	**6**
	29394327	(21)	1	0	1	1	1	1	1	0	**6**
	31267067	(22)	1	0	1	1	1	0	1	1	**6**
	33831276	(23)	1	0	1	1	1	1	0	1	**6**
	22212671	(24)	1	0	1	1	1	0	1	0	**5**
	26065405	(25)	1	0	1	1	1	0	0	1	**5**
Reasons for failure	28387389	(26)	0	1	1	1	1	1	1	1	**7**
	21532551	(27)	0	1	1	1	1	1	1	0	**6**
	23903212	(28)	0	1	1	1	1	1	1	0	**6**
	27723879	(29)	0	1	1	1	1	1	0	1	**6**
	1998766	(30)	0	1	1	1	1	1	0	0	**5**
	21283095	(31)	0	1	1	1	1	1	0	0	**5**
	27811931	(32)	0	1	1	1	1	1	0	0	**5**

Supplementary Table S5 References:

1. Wardell WM, May MS, Trimble AG. New drug development by United States pharmaceutical firms: With analyses of trends in the acquisition and origin of drug candidates, 1963–1979*. Clin Pharmacol Ther. 1982;32(4):407–17.

2. Wardell WM, DiRaddo J, Gene Trimble A. Development of new drugs originated and acquired by United States–owned pharmaceutical firms, 1963–1976. Clin Pharmacol Ther. 1980;28(2):270–7.

3. DiMasi JA. Success rates for new drugs entering clinical testing in the United States. Clin Pharmacol Ther. 1995;58(1):1–14.

4. DiMasi JA. Risks in new drug development: Approval success rates for investigational drugs. Clin Pharmacol Ther. 2001;69(5):297–307.

5. Hay M, Thomas DW, Craighead JL, Economides C, Rosenthal J. Clinical development success rates for investigational drugs. Nat Biotechnol. 2014 Jan;32(1):40–51.

6. Cook D, Brown D, Alexander R, March R, Morgan P, Satterthwaite G, et al. Lessons learned from the fate of AstraZeneca's drug pipeline: a five-dimensional framework. Nat Rev Drug Discov. 2014 Jun;13(6):419–31.

7. Morgan P, Brown DG, Lennard S, Anderton MJ, Barrett JC, Eriksson U, et al. Impact of a five-dimensional framework on R&D productivity at AstraZeneca. Nat Rev Drug Discov. 2018 Mar;17(3):167–81.

8. Bienz-Tadmor B, DiCerbo PA, Tadmor G, Lasagna L. Biopharmaceuticals and Conventional Drugs: Clinical Success Rates. Bio/Technology. 1992 May;10(5):521–5.

9. Waring MJ, Arrowsmith J, Leach AR, Leeson PD, Mandrell S, Owen RM, et al. An analysis of the attrition of drug candidates from four major pharmaceutical companies. Nat Rev Drug Discov. 2015 Jul;14(7):475–86.

10. Kola I, Landis J. Can the pharmaceutical industry reduce attrition rates? Nat Rev Drug Discov. 2004 Aug;3(8):711–6.

11. Wardell WM, Hassar M, Anavekar SN. The rate of development of new drugs in the United States, 1963 through 1975. Clin Pharmacol Ther. 1978;24(2):133–45.

12. DiMasi JA, Bryant NR, Lasagna L. New drug development in the United States from 1963 to 1990. Clin Pharmacol Ther. 1991;50(5–1):471–86.

13. DiMasi JA, Seibring MA, Lasagna L. New drug development in the United States from 1963 to 1992. Clin Pharmacol Ther. 1994;55(6):609–22.

14. DiMasi JA, Hansen RW, Grabowski HG, Lasagna L. Research and development costs for new drugs by therapeutic category. A study of the US pharmaceutical industry. PharmacoEconomics. 1995 Feb;7(2):152–69.

15. Adams CP, Brantner VV. Estimating The Cost Of New Drug Development: Is It Really $802 Million? Health Aff (Millwood). 2006 Mar;25(2):420–8.

16. DiMasi JA, Feldman L, Seckler A, Wilson A. Trends in Risks Associated With New Drug Development: Success Rates for Investigational Drugs. Clin Pharmacol Ther. 2010;87(3):272–7.

17. Sheck L, Cox C, Davis HT, Trimble AG, Wardell WM, Hansen R. Success rates in the United States drug development system. Clin Pharmacol Ther. 1984;36(5):574–83.

18. DiMasi JA, Hansen RW, Grabowski HG. The price of innovation: new estimates of drug development costs. J Health Econ. 2003 Mar 1;22(2):151–85.

19. Dahlin E, Nelson GM, Haynes M, Sargeant F. Success rates for product development strategies in new drug development. J Clin Pharm Ther. 2016;41(2):198–202.

20. Smietana K, Siatkowski M, Møller M. Trends in clinical success rates. Nat Rev Drug Discov. 2016 Jun 1;15(6):379–80.

21. Wong CH, Siah KW, Lo AW. Estimation of clinical trial success rates and related parameters. Biostatistics. 2019 Apr 1;20(2):273–86.

22. Dowden H, Munro J. Trends in clinical success rates and therapeutic focus. Nat Rev Drug Discov. 2019 May 8;18(7):495–6.

23. Yamaguchi S, Kaneko M, Narukawa M. Approval success rates of drug candidates based on target, action, modality, application, and their combinations. Clin Transl Sci. 2021;14(3):1113–22.

24. Arrowsmith J. A decade of change. Nat Rev Drug Discov. 2012 Jan 1;11(1):17–8.

25. Smietana K, Ekstrom L, Jeffery B, Møller M. Improving R&D productivity. Nat Rev Drug Discov. 2015 Jul 1;14(7):455–6.

26. Graul AI, Dulsat C, Tracy M, Cruces E. The year's new drugs & biologics 2016: Part II - Trends and highlights of an unforgettable year. Drugs Today Barc Spain 1998. 2017 Feb;53(2):117–58.

27. Arrowsmith J. Phase II failures: 2008–2010. Nat Rev Drug Discov. 2011 May 1;10(5):328–9.

28. Arrowsmith J, Miller P. Phase II and Phase III attrition rates 2011–2012. Nat Rev Drug Discov. 2013 Aug 1;12(8):569–569.

29. Hwang TJ, Carpenter D, Lauffenburger JC, Wang B, Franklin JM, Kesselheim AS. Failure of Investigational Drugs in Late-Stage Clinical Development and Publication of Trial Results. JAMA Intern Med. 2016 Dec 1;176(12):1826.

30. Rawlins MD, Jefferys DB. Study of United Kingdom product licence applications containing new active substances, 1987-9. Br Med J. 1991 Jan 26;302(6770):223–5.

31. Arrowsmith J. Phase III and submission failures: 2007–2010. Nat Rev Drug Discov. 2011 Feb 1;10(2):87–87.

32. Harrison RK. Phase II and phase III failures: 2013–2015. Nat Rev Drug Discov. 2016 Dec 1;15(12):817–8.
